# Supplementary material for: Phenotypic and Genetic Variation in Morphophysiological Traits in Huanglongbing-Affected Mandarin Hybrid Populations
Source: Plants (Basel). 2022 Dec 22;12(1):42. doi: 10.3390/plants12010042 (PMC9824356; doi:10.3390/plants12010042)
Supplement: Supplementary file 1 [file plants-12-00042-s001.zip › plants-2105003-supplementary.pdf]

**Table S1.** Selected OJIP test parameters used in this study.

| **Parameter** | **Formula** | **Meaning** |
| --- | --- | --- |
| $F_v/F_m$ or $\varphi_{Po}$ | $(F_m - F_o)/F_m$ | Maximum quantum efficiency of PSII |
| $\gamma_{RC}$ | $\frac{Chl_{RC}}{Chl_{Total}} = \frac{RC/J^{ABS}}{1 + RC/J^{ABS}} = \frac{M_o}{V_J \cdot \varphi_{Po} + M_o}$ | Probability that PSII acts as a reaction center |
| $\psi_{ET1}$ | $\frac{J_0^{TR}}{J^{ABs}} \cdot \frac{J^{ABS}}{CS} = \frac{F_m}{F_o} \cdot F_m$ | Efficiency of electron transfer from $Q_a$ to $Q_b$ |
| $\partial_{RE1}$ | $\frac{J_o^{RE1}}{J_o^{ET2}} = \frac{1- V_I}{1- V_J}$ | Efficiency of electron transfer from $Q_b$ to PSI |
| $PI_{abs}$ | $\frac{\varphi_{Po}}{1-\varphi_{Po}} \cdot \frac{\Psi_{ET1}}{1-\Psi_{ET1}} \cdot \frac{\gamma_{RC}}{1-\gamma_{RC}} \cdot \frac{\delta_{RE1}}{1-\delta_{RE1}}$ | Performance index of energy conservation from photons absorbed by PSII antenna until the reduction of PSI acceptors |

**Table S2. Diagnosis of *Candidatus* Liberibacter asiaticus infection based on qPCR *Ct* value**

| HLB Diagnosis* | HLB Positive | No HLB Found | Total | Mean | Range |
|---|---|---|---|---|---|
| First Diagnosis | 325 | 113 | 438 | 33.5±0.2 | 20.1-40.0 |
| Second Diagnosis | 208 | 236 | 444 | 36.2±4.8 | 23.4-40.0 |
| Combine Diagnosis | 362 | 108 | 444 | 33.63±0.2 | 20.1-40.0 |

* Leaf sample was collected for first diagnosis on December 24, 2019, and second was on April 20, 2020. Combine diagnosis was chosen from the lowest ct value of first and second diagnosis

**Table S3. Analysis of variance for morphological and physiological traits under different HLB score**

| Variable | Source | DF | Sum of Squares | Mean Square | F value | P value |
|---|---|---|---|---|---|---|
| LAI | Model | 8 | 193.7484 | 24.2186 | 57.1411 | <.0001 |
| | Error | 438 | 185.6409 | 0.4238 | | |
| | C.Total | 446 | 379.3893 | | | |
| Leaf area () | Model | 8 | 12698.01 | 1587.25 | 4.5052 | <.0001 |
| | Error | 439 | 154666.5 | 352.32 | | |
| | C.Total | 447 | 167364.5 | | | |
| Dry weight () | Model | 8 | 1.702692 | 0.212837 | 3.451 | 0.0007 |
| | Error | 422 | 26.02633 | 0.061674 | | |
| | C.Total | 430 | 27.72902 | | | |
| $PI_{abs}$ | Model | 8 | 13.79524 | 1.72441 | 2.0822 | 0.0362 |
| | Error | 439 | 363.5642 | 0.82816 | | |
| | C. Total | 447 | 377.3595 | | | |
| Fv/Fm | Model | 8 | 0.008395 | 0.001049 | 1.0091 | 0.4282 |
| | Error | 439 | 0.456556 | 0.00104 | | |
| | C. Total | 447 | 0.464952 | | | |
| Fv/Fo | Model | 8 | 2.7978 | 0.349725 | 1.4535 | 0.1722 |
| | Error | 439 | 105.6264 | 0.240607 | | |
| | C. Total | 447 | 108.4242 | | | |
| LMA () | Model | 8 | 4.3E-05 | 5.37E-06 | 1.4585 | 0.1704 |
| | Error | 422 | 0.001555 | 3.68E-06 | | |
| | C. Total | 430 | 0.001598 | | | |
| *Ct* value | Model | 8 | 156.2934 | 19.5367 | 0.9502 | 0.4748 |
| | Error | 428 | 8799.823 | 20.5603 | | |
| | C. Total | 436 | 8956.117 | | | |

**Table S4 Summary of Fit**

| | |
|---|---|
| RSquare | 0.496601 |
| RSquare Adj | 0.49547 |
| Root Mean Square Error | 1.161089 |
| Mean of Response | 5.872483 |
| Observations | 447 |

**Table S5 Analysis of Variance**

| Source | DF | Sum of Squares | Mean Square | F Ratio |
|---|---|---|---|---|
| Model | 1 | 591.8152 | 591.815 | 438.9909 |
| Error | 445 | 599.9163 | 1.348 | **Prob > F** |
| C. Total | 446 | 1191.7315 | | <.0001* |

**Table S6 Parameter Estimates**

| Term | Estimate | Std Error | t Ratio | Prob>\|t\| |
|---|---|---|---|---|
| Intercept | 2.111378 | 0.187722 | 11.25 | <.0001* |
| LAI | 1.2489657 | 0.059611 | 20.95 | <.0001* |

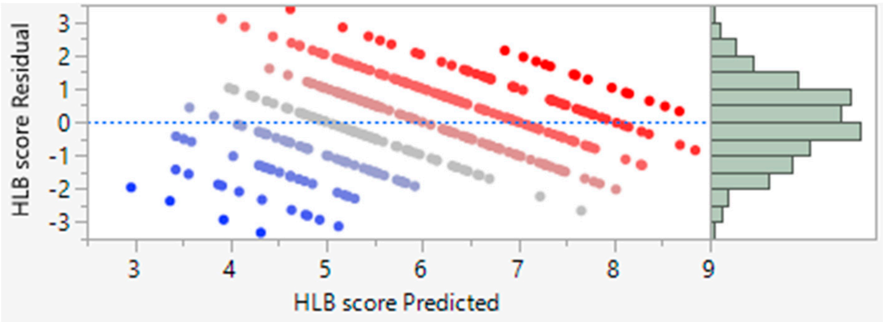


**Figure S1.** Regression analysis of HLB visual score of mandarin hybrids with distribution of HLB visual score residuals.

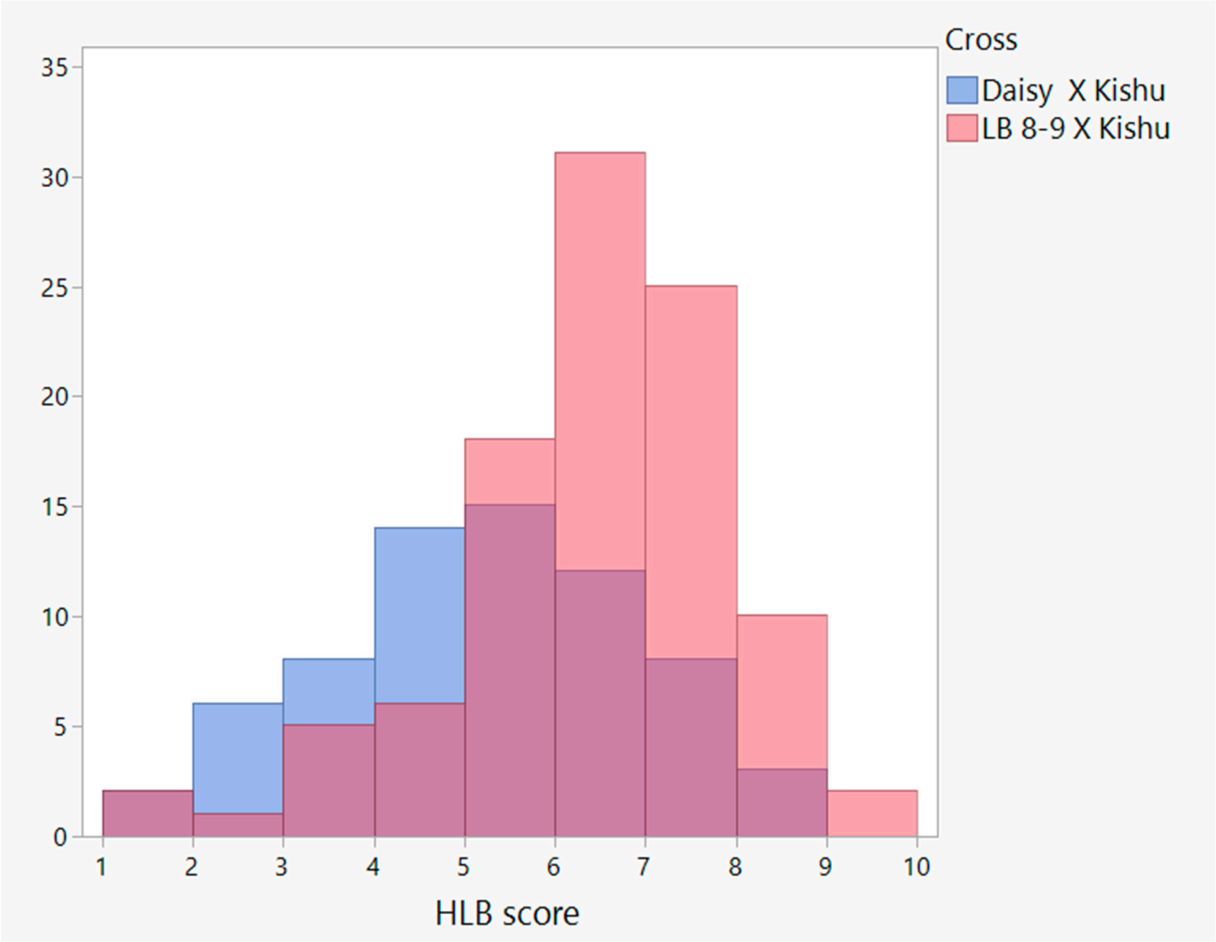


**Figure S2.** Distribution of HLB visual score in progenies of Daisy x Kishu and LB 8-9 x Kishu.
